# Geographical Affinities of the HapMap Samples

**DOI:** 10.1371/journal.pone.0004684

**Published:** 2009-03-04

**Authors:** Miao He, Jane Gitschier, Tatiana Zerjal, Peter de Knijff, Chris Tyler-Smith, Yali Xue

**Affiliations:** 1 The Wellcome Trust Sanger Institute, Wellcome Trust Genome Campus, Hinxton, United Kingdom; 2 Department of Medicine and Pediatrics, University of California San Francisco, San Francisco, California, United States of America; 3 Station de Génétique Végétale, Ferme du Moulon, Gif-sur-Yvette, France; 4 Department of Human Genetics, Center for Human and Clinical Genetics, Leiden University Medical Center, Leiden, the Netherlands; University of Montreal, Canada

## Abstract

**Background:**

The HapMap samples were collected for medical-genetic studies, but are also widely used in population-genetic and evolutionary investigations. Yet the ascertainment of the samples differs from most population-genetic studies which collect individuals who live in the same local region as their ancestors. What effects could this non-standard ascertainment have on the interpretation of HapMap results?

**Methodology/Principal Findings:**

We compared the HapMap samples with more conventionally-ascertained samples used in population- and forensic-genetic studies, including the HGDP-CEPH panel, making use of published genome-wide autosomal SNP data and Y-STR haplotypes, as well as producing new Y-STR data. We found that the HapMap samples were representative of their broad geographical regions of ancestry according to all tests applied. The YRI and JPT were indistinguishable from independent samples of Yoruba and Japanese in all ways investigated. However, both the CHB and the CEU were distinguishable from all other HGDP-CEPH populations with autosomal markers, and both showed Y-STR similarities to unusually large numbers of populations, perhaps reflecting their admixed origins.

**Conclusions/Significance:**

The CHB and JPT are readily distinguished from one another with both autosomal and Y-chromosomal markers, and results obtained after combining them into a single sample should be interpreted with caution. The CEU are better described as being of Western European ancestry than of Northern European ancestry as often reported. Both the CHB and CEU show subtle but detectable signs of admixture. Thus the YRI and JPT samples are well-suited to standard population-genetic studies, but the CHB and CEU less so.

## Introduction

The International HapMap Project was established in 2002 with the primary aim of determining the common patterns of DNA sequence variation in the human genome in order to facilitate the discovery of sequence variants that affect common diseases [1]. It was based on 270 individuals from four sources: YRI (Yoruba in Ibadan, Nigeria), CHB (Han Chinese in Beijing, China), JPT (Japanese in Tokyo, Japan) and CEU (CEPH Utah residents with ancestry from northern and western Europe). Over 3.1 million SNPs were genotyped in these samples and the patterns of linkage disequilibrium (LD) defined [2], [3]; these patterns, and the SNPs necessary to tag them have been shown to be similar in a broader set of populations, e.g. [4]. As a result, our understanding of the genetic factors influencing common diseases has accelerated considerably [5]. In addition, the availability of cell lines from these samples has allowed many additional studies to be performed, including analyses of copy number variation [6], [7] and gene expression 8, 9, while whole-genome resequencing is now under way (http://www.1000genomes.org/page.php). Moreover, the HapMap samples have been extensively used in studies searching for signals of population differentiation and natural selection, e.g. [10]–[12]. It is therefore no exaggeration to consider the HapMap samples the most intensively studied genetic samples ever.

Yet these samples, and the way in which they were collected, differ significantly from the samples used more commonly by population and evolutionary geneticists. Geneticists interested in the events that have shaped human populations over the last 50,000 years or so have usually preferred to sample individuals living in the same location as their ancestors (indigenous people), often excluding individuals whose grandparents do not all come from the same local area, or whose ancestors are known to have migrated during historical times [13]. By these criteria, the CHB and CEU samples would have been excluded. Geneticists have also generally analysed samples from different locations independently, but the CHB and JPT are often combined into a single Asian sample sometimes abbreviated ‘ASN’, e.g. [14]. What effect would the different sampling and grouping criteria introduce?

We set out to compare the HapMap samples with those more commonly used by population, evolutionary and forensic geneticists [e.g. 15], [16], [17]. We performed genomewide analyses based on published autosomal SNP genotypes [3], [18] to obtain an overall view, and supplemented these with Y-chromosomal analyses because of the uniquely powerful geographical information carried by this locus [19]. We show that, while all the HapMap samples do indeed show the general affinities expected from their ancestral origins, the paternal geographical ancestry of the CEU is slightly different from the ‘northern and western Europe’ suggested by the HapMap, and both the CHB and CEU differ in subtle ways from samples collected using more standard criteria.

## Results

**Figure 1:**
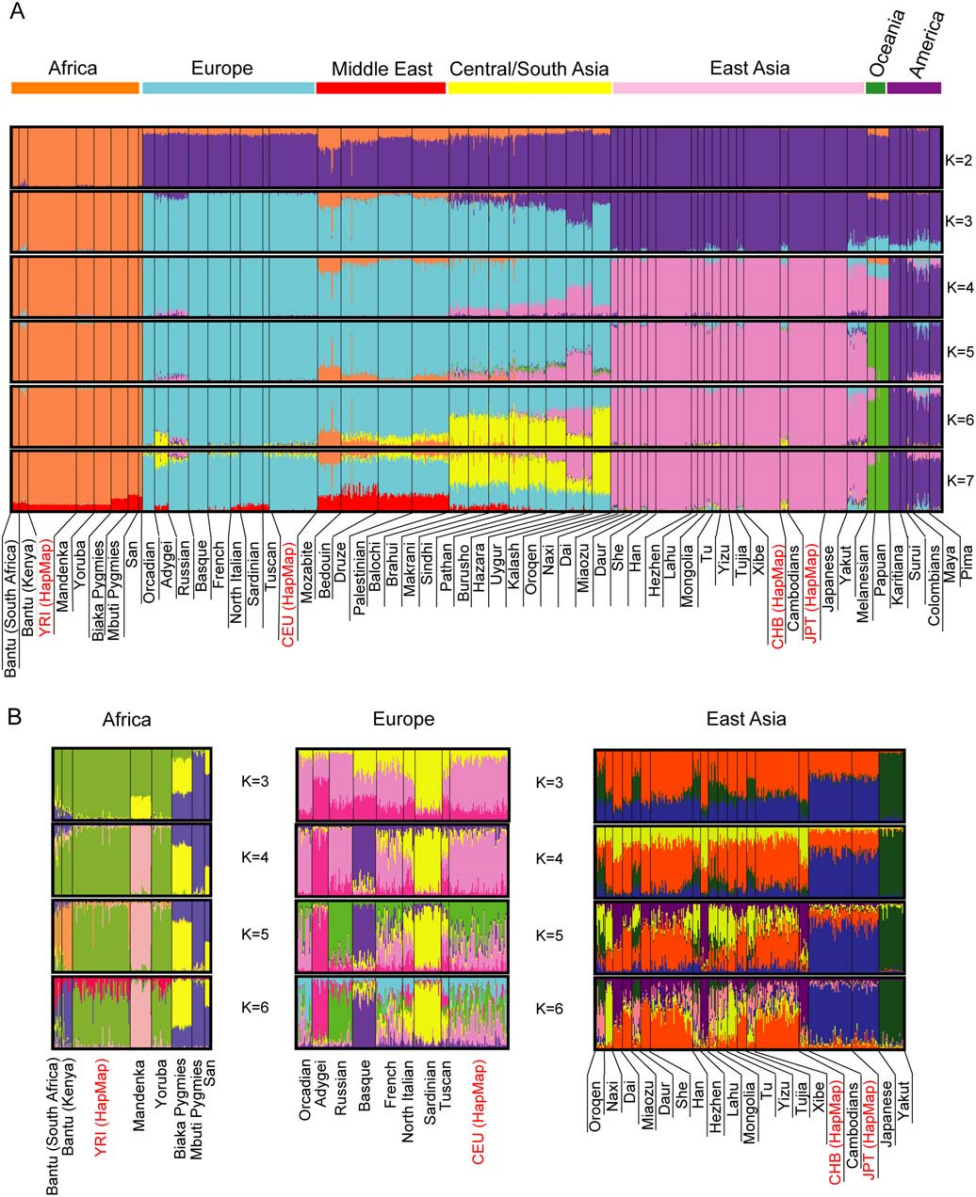
STRUCTURE analysis of the HapMap and HGDP-CEPH panels using 5,254 unlinked SNPs. A. Full dataset. B. Subsets of the panels from restricted geographical regions.

The program STRUCTURE allows individuals to be clustered on the basis of their genetic information [20]. It has previously been applied to genome-wide STR and SNP datasets from the HGDP-CEPH panel of 52 worldwide populations and identified clusters of individuals corresponding to specific geographical regions which appear to be robust and largely independent of the set of markers used [18], [21]. We performed STRUCTURE analysis on a set of genome-wide SNP genotypes from the combined HGDP-CEPH and HapMap panels using 5,254 SNPs [18] that were located ≥0.5 Mb apart and thus expected to show little LD. The STRUCTURE program requires that a number of clusters, K is specified in advance, but allows K to be varied between runs. As K was increased from 2 to 7 in different runs, clusters corresponding to finer geographical subdivisions of the world were identified, as seen when the HGDP-CEPH panel was used alone [18]. At this worldwide level of resolution, the HapMap samples always lay in the cluster expected from their ancestry (Figure 1A). We then refined the analysis by examining sub-Saharan Africa, East Asia and Europe individually (Figure 1B). In these more detailed comparisons, the YRI were still indistinguishable from the HGDP-CEPH Yoruba, and the JPT from the HGDP-CEPH Japanese (Figure 1B, Table 1). In contrast, both the CHB and CEU were distinguishable from all the HGDP-CEPH samples at higher values of K (Figure 1B). The CHB appeared most similar to the HGPD-CEPH Han or Tujia, and the CEU to the HGDP-CEPH French, but still showed visible differences in the frequency of one or more clusters (Figure 1B), and these were confirmed as statistically significant by a Mann-Whitney test after Bonferroni correction (Table 1). However, because of the limited population representation in the HGDP collection, it is possible that these samples would be more similar to other populations that had not been sampled.

**Table 1:** Comparison of frequencies of genetic clusters identified by STRUCTURE (K = 6) in HapMap samples and the most similar HGDP-CEPH sample.

Comparison	K	Cluster	p-value
YRI-Yoruba	6	1	0.108
		2	0.697
		3	0.891
		4	0.360
		5	0.235
		6	0.686
JPT-Japanese	6	1	0.460
		2	0.067
		3	0.139
		4	0.686
		5	0.367
		6	0.335
CHB-Han	6	1	0.030
		2	0.435
		3	0.086
		4	0.075
		5	0.140
		6	<0.001*
CEU-French	6	1	0.012
		2	0.005*
		3	0.045
		4	<0.001*
		5	0.021
		6	0.011

* Significant difference after Bonferroni correction for six tests.

**Figure 2:**
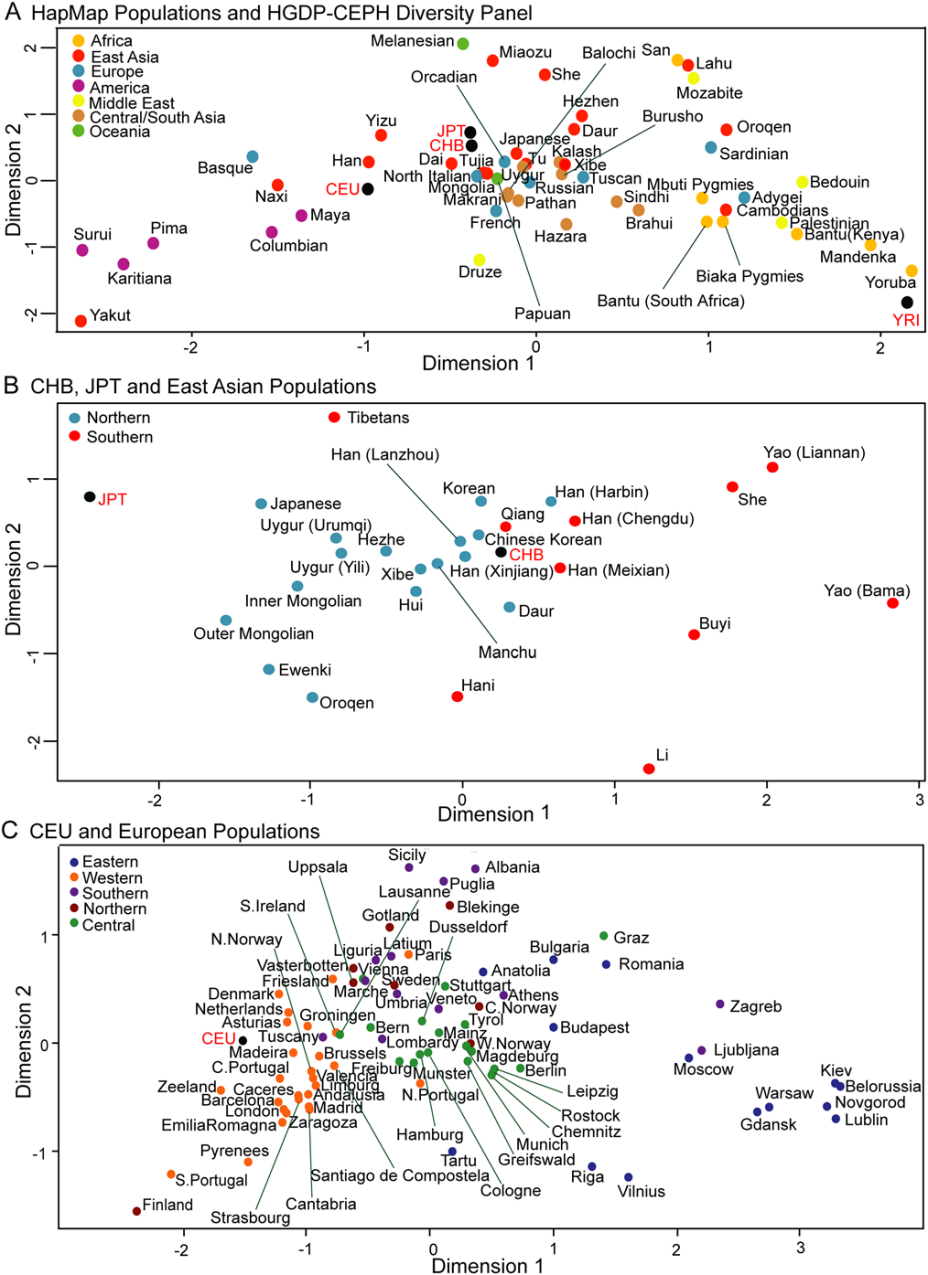
Genetic distances between populations based on Y-STR haplotypes. A. Complete HapMap and HGDP panels using 17 loci (*DYS19*, *DYS189I*, *DYS389II*, *DYS390*, *DYS391*, *DYS392*, *DYS393*, *DYS438*, *DYS439*, *DYS437*, *DYS448*, *DYS456*, *DYS458*, *DYS635*, *Y GATA H4*). B. CHB, JPT and East Asian populations using 10 loci (*DYS19*, *DYS389I*, *DYS389b*, *DYS390*, *DYS391*, *DYS392*, *DYS393*, *DYS437*, *DYS438*, *DYS439*). C. CEU and European populations using seven loci (*DYS19*, *DYS389I*, *DYS389b*, *DYS390*, *DYS391*, *DYS392*, *DYS393*).

In order to investigate their genetic relationships further, we turned to the locus that provides the highest geographical resolution, and for which large geographically-structured datasets are available: the Y chromosome. We typed the DNAs with a widely-used set of Y-STRs (Table S1), calculated population pairwise genetic distances, and compared the HapMap to the HGDP-CEPH set to provide a worldwide perspective. A Multidimensional Scaling (MDS) plot of these distances showed considerable geographical structure (Figure 2A), although not complete separation of continental regions. Nevertheless, the YRI lay closest to the HGDP-CEPH Yoruba in a cluster of African populations. The CHB and JPT lay close together near the centre of the East Asian cluster, near the Han, Yizu, Dai, Tujia and HGDP-CEPH Japanese. The CEU were located outside the main cluster of European populations, but between this cluster and the Basques who are often observed as an outlier in population-genetic studies [22]. Thus this analysis also revealed overall similarities between the HapMap samples and traditionally-ascertained samples with ancestry from the same regions.

**Figure 3:**
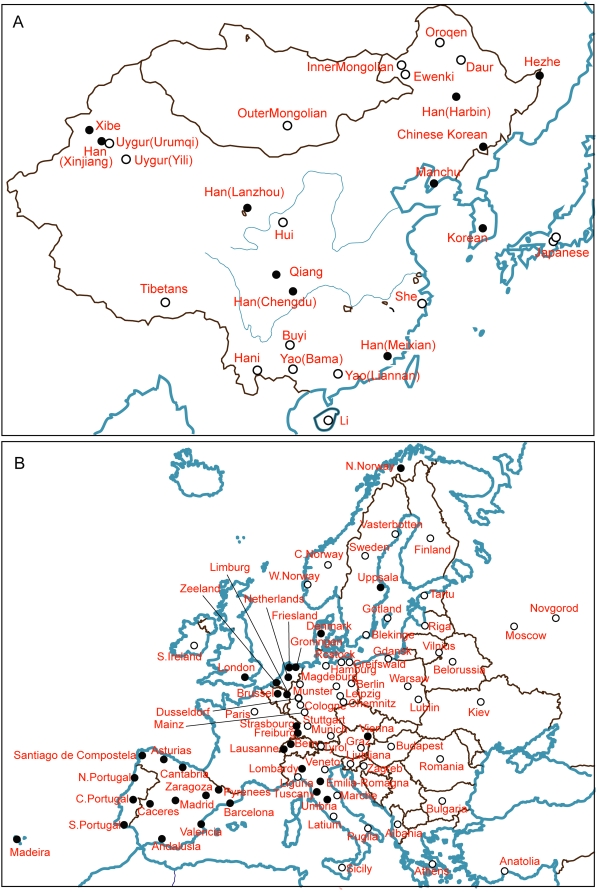
Geographical distributions of regional populations analysed with Y-STRs. A. East Asia; filled circles represent populations that are not significantly different from the CHB. B. Europe; filled circles represent populations that are not significantly different from the CEU.

It was possible to investigate these relationships further for East Asian and European samples due to the availability of additional published Y-chromosomal datasets for populations from these regions. We therefore compared the CHB and JPT to a set of 27 populations from East Asia, largely independent of the HGDP-CEPH collection [17]. The JPT again lay closest to the Japanese sample (Figure 2B), and the genetic distance between them was not significantly greater than zero, although the distance between each of the Japanese samples and all the other samples was significant (Table S2). The conclusions about the CHB were somewhat different. They lay well within the East Asian cluster. However, based on their origin in Beijing in Northern China, they would be expected to lie within the Northern cluster of East Asian populations (blue in Figure 2B). Instead, they lie at the border between the Northern and Southern clusters. Examination of the genetic distances between the CHB and the other populations revealed that they were not significantly different from 11 of the others, an unusually large number since the mean value was 3.7, SD = 3.8. The geographical distribution of these ‘similar’ samples is broad (Figure 3A), and while the Xibe and Han (Xinjiang) populations in the West are known to result from migration within the last few centuries [23], the similar populations include both Northern and Southern populations that cannot all be explained by recent migration.

The CEU were compared with a set of 81 European populations [24]. In the MDS plot they lie at the edge of the Western European cluster (Figure 2C). Interestingly, they shared with the CHB the feature of showing an unusually large number of populations with genetic distances that were not significantly greater than zero: in this case 33, compared with a mean of 15.9 and SD of 10.5. As expected from the MDS plot, the geographical distribution of these similar populations was mostly from Western Europe, with only three from Northern Europe (Figure 3B).

## Discussion

In this study we compared the HapMap samples with population samples ascertained according to more standard sampling protocols, using both autosomal and Y-chromosomal datasets. We found that they do broadly resemble other samples from the same geographical region (YRI, CHB, JPT) or with similar ancestry (CEU, Europeans). In particular, the YRI and JPT were indistinguishable from independent Yoruba and Japanese samples, respectively, by all the criteria used, but were distinct from other available samples from their regions. A detailed study of over 7,000 samples from the Japanese archipelago using >140,000 SNPs found limited substructure within this region, and also confirmed that the HapMap JPT fell into the major ‘Hondo’ cluster [25]. The CHB and CEU did not resemble in detail any of the HGDP populations when analysed with autosomal markers (Figure 1B, Table 1), but showed similarities to unusually large numbers of neighbouring populations with Y-chromosomal markers. We now consider CHB and CEU findings in more detail, and a number of implications for the use of the HapMap samples.

The lack of detailed similarity between the genome-wide autosomal genotypes of the CHB and CEU samples and the HGDP-CEPH panel could reflect the combination of high discriminatory power from such a large number of SNPs and the small number of comparison populations. In a more detailed comparison of the CEU with 2,457 individuals from 23 European populations, individual's SNP genotypes were clustered using principal component analysis [26]. Individuals from each European population generally clustered together and although the populations formed overlapping clusters, the broad North, South, East and West geographical areas of Europe were readily separated. In this analysis, the CEU were most similar to samples from the Netherlands and the UK, in agreement with the Y-chromosomal data, but in contrast were quite distinct from Spanish and Portuguese samples, which were not significantly different at the Y-chromosomal level (c.f. Figure 3B). We compared the number of samples that showed different or not different Y-chromosomal distances from the CEU in Central, Northern, Southern, Eastern and Western Europe with, in each case, the rest of Europe, using a Fisher exact test and found a striking enrichment of similar samples in Western Europe (p<0.000001) but in no other region. Some differences between a single locus and the combination of a large number of loci is unsurprising, but may also reflect the limited number of Y-STRs available for the detailed European comparison and the similarities in Y-chromosomal haplotypes throughout much of Western Europe, where haplogroup R1b predominates, being common in both Britain and Iberia [27], [28], for example. Together, these results show that the CEU, in contrast to the HapMap recommended descriptor ‘Utah residents with ancestry from northern and western Europe’ (http://www.hapmap.org/citinghapmap.html) are not appropriately described as having Northern European ancestry; Western or North-western Europe ancestry would be more accurate. A similarly detailed comparison of the CHB with additional East Asian samples would be of interest, but would require additional data, which are not yet available.

The Y-chromosomal genetic similarity of both the CHB and CEU to an unusually large number of other populations is likely to reflect their mixed origins. The CHB samples were collected from volunteers at Beijing Normal University [1],which hosts 16,000 students originating from many parts of China and including 2,000 from overseas (http://www.bnu.edu.cn/eng/about_bnu/facts_of_bnu.htm). The CEU were recruited in Utah, USA, and are descendants of Europeans whose ancestry is not well documented, but could well include more than one European country.

Finally, we emphasise one obvious point: the CHB and JPT are readily distinguished from one another with both autosomal and Y-chromosomal markers, and conclusions derived from a combined ‘ASN’ population should be interpreted with caution. For example, when we constructed an artificial mixture of equal numbers of CHB and JPT Y chromosomes, the mixture showed different characteristics from both HapMap samples and resembled five populations, including Koreans and Chinese Koreans (results not shown). While Korea is geographically intermediate, it would clearly be inappropriate to regard a HapMap sample as Korean. The HapMap study is currently being extended to additional more diverse populations in a Phase 3 (http://www.hapmap.org/index.html.en), and several of these samples also differ from conventional samples in having recently admixed and/or migrant origins, so the interpretation of the results from this phase of the project would be enhanced by including studies of the kind performed here.

## Materials and Methods

### Datasets

The genome-wide SNP genotypes of the 270 individuals in the International HapMap Project were downloaded from www.hapmap.org (Schema: rel22_NCBI_Build36), and after removing the children in the YRI and CEU samples all analyses were performed on 210 samples. Genotypes of 940 individuals from 52 populations in the HGDP-CEPH Diversity Panel (Stanford University HGDP-CEPH SNP Genotyping Data [18]) were downloaded from http://www.cephb.fr/hgdp-cephdb/. These were based on the commonly-used H952 subset [29], omitting individuals with insufficient data. Autosomal loci in common between the two datasets were then identified using a pair of Perl scripts (Script S1 and Script S2), and 5,254 loci separated by ≥0.5 Mb (and thus probably unlinked) were chosen from this list.

Y-STR data for 17 markers were generated from the HapMap and HGDP-CEPH males, again excluding the YRI and CEU sons, using the AmpFℓSTR® Yfiler® PCR amplification kit (Applied Biosystems) (*DYS19*, *DYS189I*, *DYS389II*, *DYS390*, *DYS391*, *DYS392*, *DYS393*, *DYS385I/II*, *DYS438*, *DYS439*, *DYS437*, *DYS448*, *DYS456*, *DYS458*, *DYS635 and Y GATA H4* ) [30]. Additional Y-STR data were obtained from public sources: 16 markers from 980 individuals belonging to 27 East Asian populations [17], or seven markers from over 12,700 samples from 91 locations in Europe which were downloaded from the Y-STR Haplotype Reference Database (YHRD, http://www.yhrd.org). Analyses of the different datasets used all Y-STRs except *DYS385* for the HGDP-CEPH dataset, or the subsets in common, consisting of 10 Y-STRs for the East Asian (*DYS19*, *DYS389I*, *DYS389b*, *DYS390*, *DYS391*, *DYS392*, *DYS393*, *DYS437*, *DYS438*, *DYS439*) and seven Y-STRs for the European YHRD dataset (*DYS19*, *DYS389I*, *DYS389b*, *DYS390*, *DYS391*, *DYS392*, *DYS393*).

### Statistical analyses

Population structure was investigated using the program STRUCTURE version 2.1 [20] with an admixture model. For each run, the number of clusters, K, needs to be specified in advance and values in the range 2–7 was used. Numbers of iterations in the burn-in period and MCMC replication were 4,000 and 6,000, respectively, for the runs of world-wide populations, and both 10,000 for runs of sub-regions. STRUCTURE output was processed with CLUMPP [31] and distruct (http://rosenberglab.bioinformatics.med.umich.edu/distruct.html). Cluster frequencies were compared between pairs of populations using a Mann-Whitney U test implemented in SPSS 16.0. Population pairwise genetic distances (*Φ*_ST_ values) were calculated from Y-STR haplotypes using the Arlequin package (http://lgb.unige.ch/arlequin/) and their significance was assessed from 1,000 bootstrap simulations, except for the European dataset where that these calculations did not reach completion and *R*_ST_ values were used. MDS analysis of population pairwise distances was carried out using SPSS 16.0. RSQ and stress values were: HGDP, 0.81 and 0.23; East Asia, 0.89 and 0.17; Europe, 0.95 and 0.13.

### Electronic database information

1000 Genomes: http://www.1000genomes.org/page.php

Arlequin: http://lgb.unige.ch/arlequin/

Distruct: http://rosenberglab.bioinformatics.med.umich.edu/distruct.html

HapMap data: www.hapmap.org

HGDP-CEPH data: http://www.cephb.fr/hgdp-cephdb/

STRUCTURE: http://pritch.bsd.uchicago.edu/structure.html

YHRD: http://www.yhrd.org

## Supporting Information

Table S1 HapMap YSTR haplotypes
(0.04 MB XLS)

Table S2 Population pairwise comparisons
(0.18 MB XLS)

Script S1 Perl script 1
(0.00 MB TXT)

Script S2 Perl script 2
(0.00 MB TXT)
